# Announcement: 5th International Symposium on Applied Bioinorganic Chemistry

**DOI:** 10.1155/MBD.1998.175

**Published:** 1998


					5TH INTERNATIONAL SYMPOSIUM ON
APPLIED BIOINORGANIC CHEMISTRY
CORFU, GREECE, April 13 17 1999
Scope
The 5th International Symposium on Applied Bioinorganic Chemistry, 5th ISABC, is the 5th in a
series of similar symposia that were first initiated by Kui Wang of Beijing Medical University and John
Webb of Murdoch University, Perth, Australia.
Though the field of Bioinorganic Chemistry has progressed considerably over the past years, the
significance of its applications to human welfare and development have not yet been widely
appreciated. Besides the International Conference of Bioinorganic Chemistry and the European
Conference of Bioinorganic Chemistry, each one held every two years, the International Symposia
on Applied Bioinorganic Chemistry were initiated to provide current perspectives on the science,
besides the established applications of Bioinorganic Chemistry and to stimulate developments that
would lead to further applications. It aims therefore to fill up the gap and emphasize mainly the
possible applications of Bioinorganic Chemistry. With this purpose, the 5th International Symposium
on Applied Bioinorganic Chemistry will involve Plenary and Session Lectures in the following topics:
1) Metalloenzymes
2) Biomaterials
3) Metal Based Drugs
4) Metals in the Environment and Metal Toxicology
5) Bioelectronics
6) Spectroscopic Applications
7) Model Compounds
8) Metal Speciation
Many experts of international reputation have accepted to take part to the 5th ISABC.
Consequently, the participation of young scientists is strongly encouraged, so that they become
aware of all possible modern applications of the field and in their turn contribute to its further
development.
Venue and accommodation
The 5th ISABC will be held in the beautiful island of Corfu, located in the Northwest of Greece,
approximately 1.5 hours by boat from the closest continental port of Igoumenitsa. The period of the
Symposium, 13 -17 April, follows the Greek Easter Sunday (April 11, 1999) and may be of interest
for those wishing to come a little earlier on this occasion. The average temperature in April in Corfu is
expected to be about 20 C.
In addition, Satellite Symposia on recent developments in Thalassemia (I) and modern NMR & EPR
spectroscopic applications (11) will be organized at the University of Ioannina, on 19 & 20 of April,
1999. Ioannina is a town of approximately 120000 inhabitants, located 92 Km west of the port of
Igoumenitsa (the closest to Corfou).
The symposium will be held at the hotel CHANDRIS in Corfu and the official language will be English.
Aproximate fare for half board pension per day, will be:
Hotel CHANDRIS****
Half board, single room: 65 US $ Half board, double room: 50 US $
175
Other convenient and comfortable hotels located at the seashore at a walking distance from lecture
halls will also be available. Details and information on hotel accomodation, air and sea connections
will be given in the second circular.
Submission of Abstracts for Contributed Papers
Papers related to the general field of applied Bioinorganic Chemistry are welcome.
Abstract forms and instructions will be given in the Second Circular.
Registration fees will be 220 US $ for active participants and 50 US $ for accompanying
persons. To encourage attendance by young scientists, registration fees will be reduced to only 75
US $ for graduate students presenting oral papers or posters.
Additional 80 US $ registration fees will be required for the attendance of the Satellite Symposia
from active participants and will be free for graduate students.
The 5th ISABC is organized by the University of Ioannina
Chairman: Prof. Nick HADJILIADIS
International Organizing Committee
I. BERTINI (Italy) I.S. BUTLER (Canada) J. BUFFLE (Switzerland) S.BERNS-PRICE (Australia)
D. COUCOUVANIS (USA) I. GEROTHANASSIS (Greece) M. GIELEN (Belgium)
F. GONZALES-VILCHEZ (Spain) G.E. JACKSON (South Africa) L. N. JI (China) N. KATSAROS
(Greece) V.G. KUMAR DAS (Malaysia) E. KIMURA (Japan) B. LIPPERT (Germany) G. NATILE
(Italy) E. RATILA (Philippines) J. REEDIJK (The Netherlands) H. SIGEL (Switzerland) AG.
SYKES (UK) J.P. TUCHAGUE (France) K. WANG (China) J. WEBB (Australia) D.R. WILLIAMS
(UK)
Local Organizing Committee
Nick HADJILIADIS, Univ. of Ioannina (Chairman)
Nick KATSAROS, NCSR "DEMOKRITOS" Athens (Vice Chairman) Spyros PERLEPES, Univ. of
Patras Dimitris KESSISSOGLOU, Univ. of Thessaloniki. Athanassios COUTSOLELOS, Univ. of
Crete Costas METHENITIS, Univ. of Athens Theophile THEOPHANIDES, N.T. Univ. of Athens
Ioannis GEROTHANASSIS, Univ. of Ioannina John TSANGARIS, Univ. of Ioannina Themistoklis
KABANOS, Univ. of Ioannina Dimitra KOVALA-DEMERTZI, Univ. of Ioannina John
PLAKATOURAS, Univ. of Ioannina Maria LOULOUDI, Univ. of Ioannina Sotiris HADJIKAKOU,
Univ. of Ioannina
Invited Speakers (will include):
S.LIPPARD (USA) J.REEDIJK (The Netherlands) I.BERTINI (Italy) D. WILLIAMS (UK) V.
PECORRARO (USA) D. COUCOUVANIS (USA) V. PAVONE (Italy) B. SARKAR (Canada) N.
HADJILIADIS (Greece) M. STILLMAN (Canada) H. SIGEL (Switzerland) E.KIMURA (Japan) K.
TIMMIS (Germany) D. MANSUY (France) K. KASPRZAK (USA) K. WANG (China) L. CASELLA
(Italy) G. SYKES (UK) T. KISS (Hungary) B. LIPPERT (Germany) T. THEOPHANIDES (Greece)
G.E. JACKSON (S. Africa) P. SADLER (UK) I.S. BUTLER (Canada) R. BARBUCCI (Italy) J.
WEBB (AUSTRALIA) M.GIELEN (Belgium) I. GEROTHANASSIS (Greece) I. SOVAGO (Hungary)
F. GONZALES-VILCHEZ (Spain) D. KESSISSOGLOU, (Greece) G. NATILE (Italy) H.
KOZLOWSKI (Poland) J.P. TUCHAGUE (France) L.N. Jl. (China)
Possible Participation of J.M. LEHN (France, Nobel Prize)
Conference secretariat
a) Dr.MariaLOULOUDI .Tel.: +30 651 98 418 or 98 429
Fax: +30 651 44 831 e-mail: mlouloud@cc.uoi.gr
b) Dr. Sotiris HADJIKAKOU Tel.: +30 651 98 374
Fax: +30 651 44 825 e-mail" shadjika@cc.uoi.gr
5th SABC Secretariat
University of Ioannina, Department of Chemistry,
Laboratory of Inorganic & General Chemistry
GR-451 10, Ioannina, Greece
176

				

